# Cannabinoids detected in exhaled breath condensate after cannabis use

**DOI:** 10.1088/1752-7163/ad6347

**Published:** 2024-07-23

**Authors:** Jennifer L Berry, Ashley Brooks-Russell, Cheryle N Beuning, Sarah A Limbacher, Tara M Lovestead, Kavita M Jeerage

**Affiliations:** 1 Applied Chemical and Materials Division, National Institute of Standards and Technology, 325 Broadway, Boulder, CO, United States of America; 2 Colorado School of Public Health, University of Colorado Anschutz Medical, 13001 E. 17th Place, Aurora, CO, United States of America

**Keywords:** cannabis, forensic toxicology, Δ^9^-tetrahydrocannabinol (THC)

## Abstract

Cannabinoids can be detected in breath after cannabis use, but different breath matrices need to be explored as studies to date with filter-based devices that collect breath aerosols have not demonstrated that breath-based measurements can reliably identify recent cannabis use. Exhaled breath condensate (EBC) is an unexplored aqueous breath matrix that contains condensed volatile compounds and water vapor in addition to aerosols. EBC was collected from participants both before and at two time points (0.7 ± 0.2 h and 1.7 ± 0.3 h) after observed cannabis use. Eleven different cannabinoids were monitored with liquid chromatography tandem mass spectrometry. Five different cannabinoids, including Δ^9^-tetrahydrocannabinol (THC), were detected in EBC collected from cannabis users. THC was detected in some EBC samples before cannabis use, despite the requested abstinence period. THC was detected in all EBC samples collected at 0.7 h post use and decreased for all participants at 1.7 h. Non-THC cannabinoids were only detected after cannabis use. THC concentrations in EBC samples collected at 0.7 h showed no trend with sample metrics like mass or number of breaths. EBC sampling devices deserve further investigation with respect to modes of cannabis use (e.g, edibles), post use time points, and optimization of cannabinoid recovery.

## 1. Introduction

The prevalence of cannabis use is increasing, coinciding with the increasing legalization of adult use in U.S. states [1, 2]. This poses the challenge for public health and safety of detecting recent cannabis use on roadways and in safety-sensitive work settings, because cannabis use can lead to impairment [3–5]. However, a non-invasive, portable, and accurate test for cannabis use has been elusive. Breath analysis has several mature applications, including clinically to identify or monitor disease. Furthermore, the relationship between the concentration of alcohol in breath and intoxication is widely used in the forensic field [6]. Based on the widespread acceptance of the alcohol breathalyzer by law enforcement, the legal community, and the public, reliable breath-based measurements could find widespread use in the detection of recent cannabis use.

Detecting cannabis in breath has proven substantially more challenging than ethanol (alcohol). Research to date has focused on Δ^9^-tetrahydrocannabinol (THC), the main psychoactive component of cannabis. THC and other cannabinoids have a much lower vapor pressure than ethanol [7] and are expected to be exhaled in breath aerosols. Therefore, researchers have focused on collecting breath aerosols with filter-based devices through interception, electrostatic forces, and impaction. Interception filters collect aerosols following along the fluid flowlines onto filter fibers. Electrostatic filters attract charged aerosols with static electricity, and this mechanism is often employed simultaneously with interception. Impaction filters collect aerosols by exploiting the high moment of inertia that causes aerosols to deviate from the fluid flowlines, hitting surfaces that gas streams can go around. While a filter’s material properties and geometry may be selected to promote a particular mode of action, multiple mechanisms may be important, depending on aerosol dimensions and fluid dynamics. An optimal collection strategy has not yet been identified.

Different devices have been explored to collect exhaled cannabinoids after known cannabis use events. One commonly used device is the SensAbues device, which uses an electrostatic filter [8–13]. Despite using the same device and often the same breath sampling protocol, THC concentrations at 1.0–1.5 h after cannabis use vary over four orders of magnitude from ∼0.05 ng/device to >100 ng/device. The time frame that THC is detectable in breath also varies between studies, with some reporting ‘not detected’ for most participants roughly 1 h after use [12] and others reporting detection at or after 3 h [9, 10, 13]. The Hounds Lab electrostatic filter device [14] and BreathExplor impaction filter device [15, 16] have been investigated as well, expanding the THC detection range to as low as 0.01 ng/device at 1 h after cannabis use. Breath THC research is in the very early stages and there are no standardized procedures. Differences in breath sampling devices, breath sampling protocols, and processing may explain the large range of results and there is almost certainly within device variability due to the aerosol capture process as well. For example, simulations of an impaction filter showed the efficiency of aerosol collection depends on fluid velocity [17]. Devices that rely on other modes of action might not have the same challenges as filter-based devices.

Exhaled breath condensate (EBC) has not yet been explored in the context of recent cannabis use. EBC is collected by condensing exhaled breath components with pre-chilled metal collars, cooling material such as dry ice pellets [18], or active cooling systems [19], providing an aqueous sample that contains condensed water vapor, water-soluble volatile organic compounds, semi-volatiles, non-volatiles, and aerosols trapped by sedimentation. EBC was used historically to explore respiratory diseases like asthma [20, 21] and recently was used to detect oxylipin levels in Covid-19 patients [22]. EBC has also been used to detect metabolites resulting from infused opioid drugs [23] and has the potential for collecting exhaled cannabinoids as well. EBC has an advantage over aerosol-only methods because of the additional breath components contained in this matrix. While THC is unlikely to be fully gaseous at breath relevant conditions due to its low vapor pressure, a non-negligible fraction may be present as a vapor based on partitioning theory [24, 25]. EBC devices do not require any assumptions about THC phase state because both vapor and aerosols are collected. Determining if THC and other cannabinoids can be recovered from this unexplored matrix would lay the groundwork for future studies to explore if EBC is a more robust breath matrix with which to determine recent cannabis use.

The goal of this proof-of-concept study was to determine if THC and 10 other cannabinoids could be detected in aqueous EBC after observed cannabis use. EBC was collected immediately before and at two time points after cannabis inhalation (0.7 and 1.7 h) that fall within the driving impairment window as assessed by decreases in the composite drive score [26]. This study shows the first ever measurements of cannabinoids in EBC and is the first exploration into cannabinoids in an aqueous breath matrix.

## 2. Materials and methods

### 2.1. Study participants

The data presented are initial findings from a larger study with a primary goal of studying cannabis impairment. The Colorado Multiple Institutional Review Board (COMIRB protocol 20-0949) and National Institute of Standards and Technology Institutional Review Board (NIST IRB protocol MML-2022-0396) approved study procedures. Participants came to an off-campus research site to complete data collection and were asked to abstain from cannabis inhalation for at least 8 h and from cannabis ingestion for at least 12 h before the start of their visit. Participants were asked to bring their own cannabis labeled with THC concentration and containing less than 2% cannabidiol (CBD) from a licensed Colorado dispensary and were given 15 min to consume their cannabis *ad libitum*. Cannabis inhalation (smoking flower or vaping concentrate) occurred at the research site in a designated room with a ventilation system to remove smoke and vapors. Participants who did not use cannabis were invited to take a break for 15 min or continue data collection. EBC samples were collected from 12 participants who inhaled cannabis and 2 control participants who did not use cannabis. Due to the limited number of participants in this study, no statistical comparisons will be presented here. Future publications will address comparisons between cannabis administration route, cannabis potency, self-reported cannabis use, and blood cannabinoid concentrations with a larger dataset.

### 2.2. EBC collection

EBC was collected with the RTube (Respiratory Research) in a different room at the research site than where cannabis was inhaled. This device utilizes a metal collar to chill the collection chamber and thereby condense exhaled breath components. The collar (stored at −80 °C) was placed over the collection chamber immediately prior to each sample collection, with a fresh collar used for each sample. Samples were collected before cannabis use and at two time points after cannabis use, 0.7 ± 0.2 h (44 ± 9 min) and 1.7 ± 0.3 h (104 ± 10 min). All participants were read a standard script with instructions. Participants breathed through the device for 5 min while using a deep breathing maneuver to increase aerosol production [27]. After collection, samples were stored at −80 °C until analysis.

### 2.3. EBC analysis

EBC samples were thawed at room temperature, removed from the collection chamber with a glass pipet, and placed into a silanized glass vial. EBC from each RTube was then re-frozen at −80 °C and lyophilized to dryness. The lyophilized solids were reconstituted with 100 *μ*l of 35% water/65% methanol that contained nominally 10 ng g^−1^ of each internal standard and transferred to an autosampler vial with a silanized glass insert. During lyophilization, each participant’s samples were covered with a lint-free tissue and split between four different lyophilization containers based on mass. Control experiments were performed by lyophilizing THC-spiked samples with blank samples (EBC samples from known non-users). Without the tissue covering, THC from the spiked sample contaminated the blank sample, whereas with the tissue covering, no cross-contamination occurred.

THC, Δ^8^-tetrahydrocannabinol (Δ^8^-THC), Δ^10^-tetrahydrocannabinol (Δ^10^-THC), CBD, cannabinol (CBN), cannabigerol (CBG), cannabichromene (CBC), tetrahydrocannabivarin (THCV), tetrahydrocannabinolic acid (THCA), cannabigerolic acid (CBGA), and 11-nor-9-carboxy-delta-9-tetrahydrocannabinol (THC-COOH) were separated by liquid chromatography and quantified by tandem mass spectrometry (LC-MS/MS) with 20 *μ*l injections. Information on the instrumental method can be found in the supplemental materials, which includes the chromatographic separation (supplemental figure 1), mass spectrometry parameters (supplemental table 1), limit of detection and lower limit of quantification (supplemental figure 2), linear dynamic range (supplemental table 2), accuracy of quality control replicates (supplemental figure 3 and supplemental figure 4), and representative chromatograms from authentic breath samples (supplemental figure 5). The linear dynamic range of the method is demonstrated from nominally 250 to 0.04 ng g^−1^ with an accuracy within ±15% for all analytes, with the exception of THC–COOH, which had a linear dynamic range to 0.08 ng g^−1^.

For authentic breath analysis, nine calibrators were prepared from nominally 150 to 0.1 ng g^−1^ and the calibration curve used 1/*x* ^2^ weighting (0.98 < *R* ^2^ < 1.00 for all analytes). Analytes were positively identified by using their qualifier-to-quantifier ratio (±20%) and retention time compared to internal standards (⩽0.05 min). Analytes were quantified if they were ⩾0.04 ng g^−1^ based on the linear dynamic range established with standard mixtures. Peaks below this concentration that met all other criteria (qualifier-to-quantifier ratio and retention time) were designated as trace (tr) concentrations. Concentrations calculated in ng g^−1^ for authentic samples were converted to ng/device by multiplying the concentration by the mass of the reconstituted lyophilized sample. Measured reconstituted masses averaged 0.086 g ± 0.001 g, so the lower limit of quantification was approximately 0.003 ng/device. Representative chromatograms for select calibrators and authentic samples can be found in supplemental figures 1 and 5, respectively.

## 3. Results

### 3.1. Cannabinoids in EBC

EBC samples from 12 participants who inhaled cannabis and two non-user participants were analyzed. The two non-users had no detectable THC or other cannabinoids in their samples. This is an expected result because the non-user participants were not simply regular cannabis users assigned as a control group, but rather participants without any exposure to cannabis in their daily life. Their samples provide important confirmation that residual cannabinoids at the research site (from previous participants’ smoking or vaping) did not contaminate breath samples. Therefore, any cannabinoids detected in the breath of participants who used cannabis can be assumed to be from cannabis use and not environmental contamination.

For the participants who inhaled cannabis, THC was detected in 5 of 12 participants prior to use and no other cannabinoids were detected (table 1). At 0.7 h, THC was detected in all participants, with P10 having trace levels. At 0.7 h, CBN was detected in 6 of 12 participants, with P02 having trace levels. CBG was simultaneously detected in 5 of these 6 participants. At 0.7 h, THCA was quantifiable in one participant and THCV was detected as trace from a different participant. By 1.7 h, THC concentrations had decreased for all participants who used cannabis. When detected, CBN concentrations also decreased, and no other cannabinoids were detected.

**Table 1.** Cannabinoids (ng/device) detected pre-use and 0.7 ± 0.2 h or 1.7 ± 0.3 h after cannabis use. Non-users were not included in the table as no cannabinoids were detected at any time points.

ID	Pre-use					0.7 ± 0.2 h					1.7 ± 0.3 h				
	THC	CBN	THCA	THCV	CBG	THC	CBN	THCA	THCV	CBG	THC	CBN	THCA	THCV	CBG
P01	0.003					0.085	0.007			0.006	0.011	*tr*			
P02						0.032	*tr*			0.003	0.006				
P03						0.017					0.006				
P04						0.011									
P05	0.004					0.015					0.007				
P06						0.113	0.005	0.011		0.004	0.025	*tr*			
P07						0.435	0.042			0.022	0.010	*tr*			
P08	0.004					0.014					0.005				
P09	0.008					1.250	0.011		*tr*	0.024	0.003				
P10	*tr*					*tr*									
P11						0.014	0.004				0.008				
P12						0.006									

### 3.2. Reliability of EBC as a matrix

**Figure 1.**
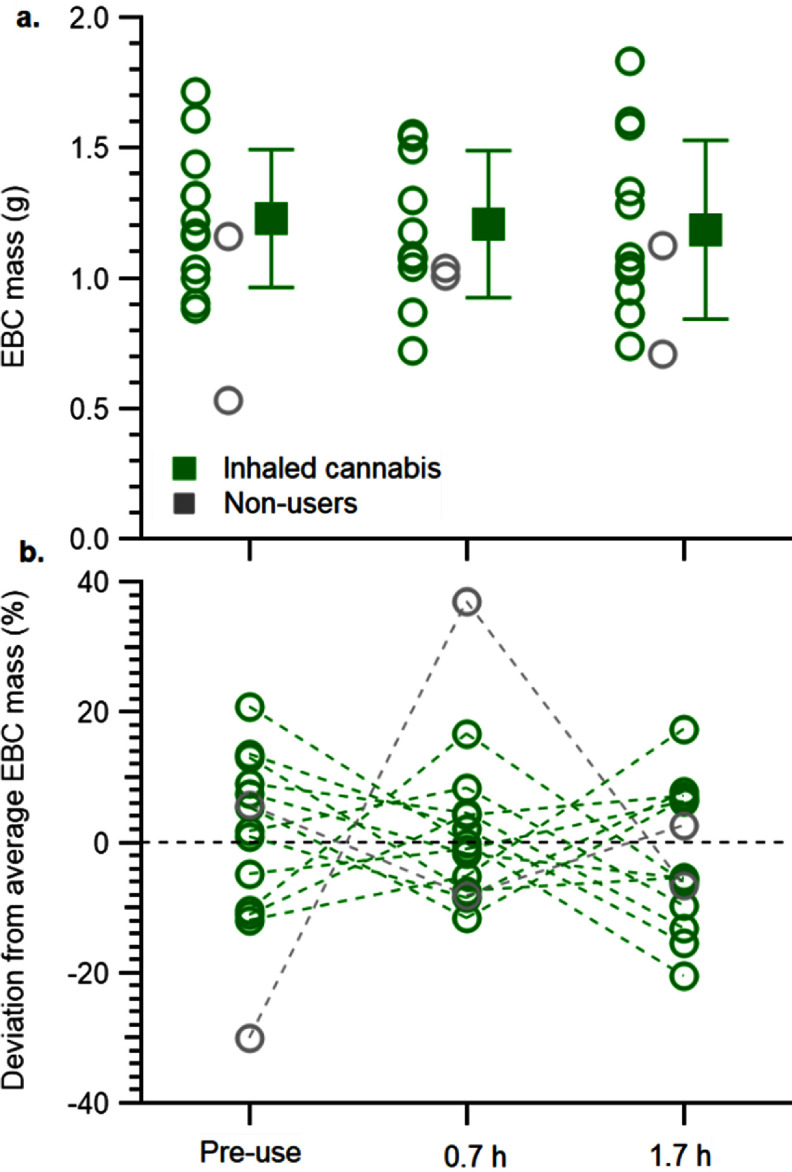
(a) EBC mass with averages (squares) and standard deviations at each time point and (b) percent deviation from average EBC mass from individual participants who inhaled cannabis (n = 12) and non-users (n = 2). Dotted lines connect the same participant at different time points.

Unlike exhaled breath aerosols, which do not appreciably change the mass of the collection device, the mass of EBC can be quantified. EBC mass was compared between and within participants at each time point. EBC mass was expected to vary between participants, because exhalation volume varies dramatically by age and sex, and also between time points. We were specifically concerned that EBC masses collected after cannabis use would be significantly lower due to the challenge of providing a sample when presumably impaired. EBC masses were all greater than 0.5 g. The average EBC mass obtained from participants who used cannabis (figure 1(a)) was not significantly different at each time point. When all samples are considered, the average EBC mass has a coefficient of variance of 28%. Therefore, we also examined each participant individually, by comparing EBC mass at each timepoint to the average EBC mass for that participant (figure 1(b)). Most participants deviated from their respective average EBC mass by less than 20% (figure 1(b)), except for one participant that inhaled cannabis and one non-user.

**Figure 2.**
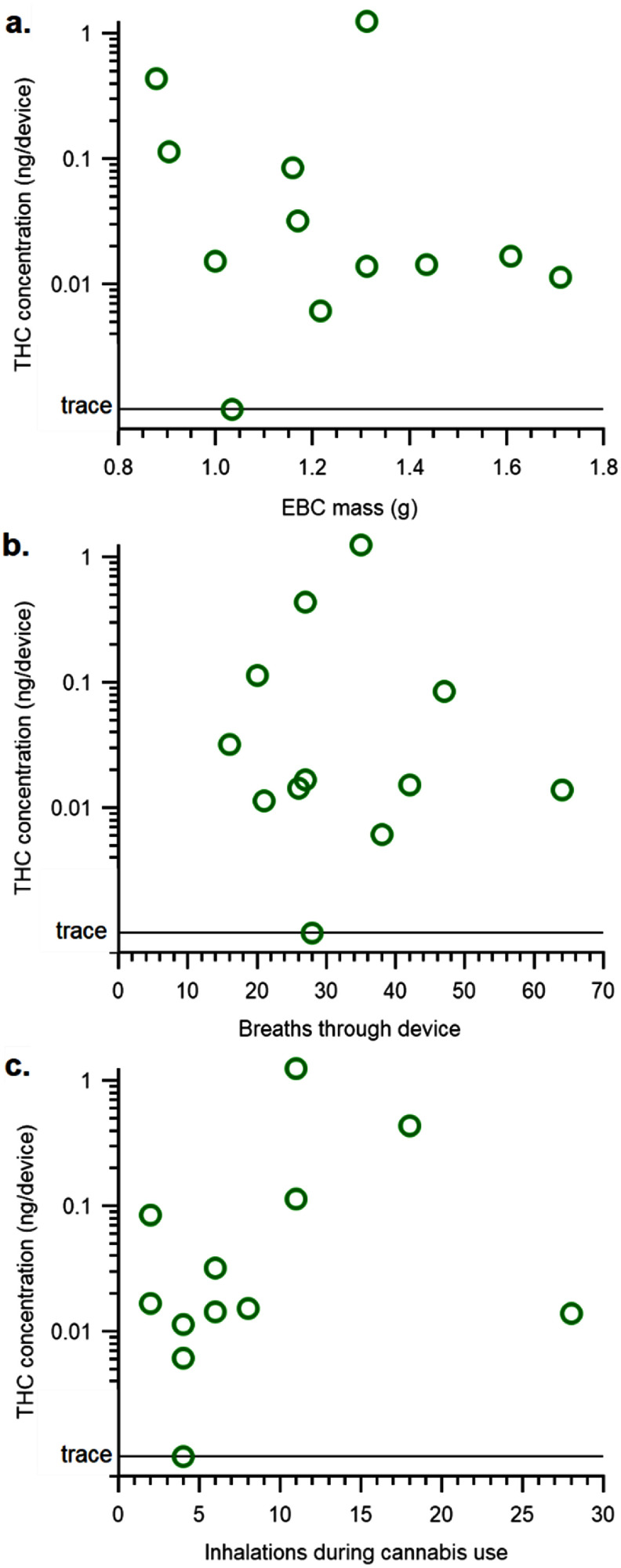
THC concentration (ng/device) at 0.7 h from participants who inhaled cannabis compared to (a) the EBC mass, (b) the number of breaths through the device, and (c) the number of inhalations from the self-supplied cannabis product.

The correlation of THC concentration at 0.7 h with different metrics was also investigated. THC concentration showed no obvious trends with respect to two sampling metrics (figures 2(a) and (b)). Each participant in this study provided their own cannabis product and presumably inhaled enough to reach their desired intoxication level, with the number of observed inhalations ranging from 2 to 28. THC concentration also showed no trend with this metric (figure 2(c)). P09 had the highest THC concentration, but intermediate sampling and cannabis use metrics. P10 was not an outlier with respect to sampling metrics, indicating that the trace THC concentration did not result from an unusual collection. P11 appeared to have a shallow breathing pattern, but this participant’s EBC mass was typical. Additionally, THC concentration showed no trend with freezer temperature, which is the presumed temperature of the collection chamber during sampling, or with the length of time each sample was stored in the collection chamber before processing and analysis (data not shown).

## 4. Discussion

Five different cannabinoids—THC, CBN, CBG, THCV, and THCA—were detected in EBC samples from cannabis users during the expected timeframe of impairment after cannabis use. The non-THC cannabinoids detected here are consistent with past studies. Roughly 50% of participants in our study had CBN and CBG at 0.7 h. CBN has been detected in the breath of some participants after cannabis use by all studies that have monitored it [8, 13–15]. CBG has only been monitored in one previous study [13]. Wurz and DeGregorio [13] detected CBN, CBG, and THCV from approximately 90% of participants within the first hour after cannabis use, but their results had not been replicated until this work, though we had lower detection rates. We also detected THCA in one participant. This cannabinoid has not been previously detected in the breath of cannabis users. Lower detection rates may be a consequence of cannabinoid recovery. All past studies used an organic rinse to extract cannabinoids from breath devices into solution, which is a logical choice given the lipophilicity of cannabinoids. Though it was not possible to use an organic rinse here, this could be done with other device materials. This study shows the first ever measurements of cannabinoids in a breath matrix that is aqueous and, furthermore, the collection strategy used here does not require the assumption that THC is primarily carried by aerosols.

THC concentrations in breath at roughly 1-h post use span five orders of magnitude across published studies [15]. It is worth noting that detection limits play a role in detection ranges as well. For example, Hubbard *et al* [12] and Fitzgerald *et al* [28] had a lower limit of quantification of 0.08 ng/device, which falls close to the average THC concentrations from Lynch *et al* [14] and Jeerage *et al* [15]. This suggests that THC could have been detected in many more of their participants with more sensitive methods. THC has also been detected in the breath of cannabis users prior to cannabis use despite requested abstinence in previous studies [8, 12–15]. Despite the fact that noncompliance cannot be ruled out in self-reported abstinence studies, most recent studies have relied on self-reported abstinence ranging from 12 to 48 h and report some detection of THC in breath prior to use [12–15]. If the measured THC quantities represent the participant’s baseline levels, a single breath THC measurement could only be used to determine recent use if a THC concentration associated with recent use can be established, akin to a *per se* limit. No one has proposed a ‘cutoff’ THC concentration for breath measurements and attempts to identify cutoff THC concentrations in blood or oral fluid have been found to be insufficient to determine recent use or impaired driving [12, 28, 29]. Based on limited studies to date, non-THC cannabinoids such as CBN and CBG are only detected after cannabis use. Therefore, they could potentially be used in conjunction with THC detection to show that cannabis has been used very recently. An example from another matrix is that molar metabolite ratios of cannabinoids in blood were better indicators of recent cannabis smoking than whole blood THC alone [30].

**Figure 3.**
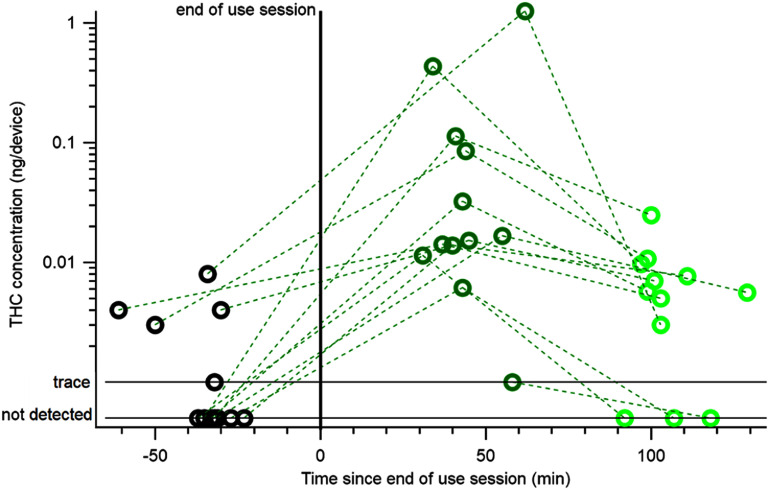
THC concentration (ng/device) from participants who inhaled cannabis. Dotted lines connect the same participant at different time points (black is pre-use, dark green is first post use time point, and light green is second post use time point). This figure shows that THC concentrations increase after cannabis use and decrease during the expected cannabis impairment window.

In this study, THC was detected in EBC at 0.7 h from every participant that inhaled cannabis (figure 3) and the THC concentration at this time point was always higher than the THC concentration measured prior to cannabis use (the presumed baseline). THC concentration decreased for every participant by 1.7 h. The increase from baseline to the first post use time point, combined with the decrease at the second time point suggests that most of the THC collected is from a recent use event and not from systemic cannabinoids. EBC components derived from the lungs are diluted to a variable degree by condensed water and this dilution can be accessed via additional measurements [21]. Here, the sample-to-sample differences in EBC mass are small compared to THC concentration differences, meaning that normalization by EBC mass does not change figure 3 and its implications. Specifically, the decrease in THC concentration from 0.7 to 1.7 h after cannabis use suggests that multiple measurements across time might be a better indication of recent use than measurements at a single time point. Future studies should investigate the relationship between THC elimination and anthropometric measures (height, weight), sex, and age, which are used for alcohol calculations in forensic toxicology.

## 5. Conclusions

EBC contains both breath aerosols and volatile organic compounds and has never been explored with respect to cannabis use. The measurements described here show that EBC is a promising breath matrix with which to collect cannabinoids after cannabis inhalation. Participants who smoked flower or vaped concentrates to their desired level of intoxication were able to complete the EBC sampling protocol within the first hour after cannabis use, including safely handling the chilled metal collar. EBC mass varied by 20% or less within participants. THC was detected in all EBC samples collected 0.7 h after cannabis use and decreased in concentration by 1.7 h. THC concentrations and the detection of non-THC cannabinoids including CBN and CBG were consistent with past studies using different breath collection devices. As a THC ‘cutoff’ in breath to determine recent use has not been established, multiple measurements of THC concentration across time might be a better indication of recent use and should be investigated further. Since non-THC cannabinoids were only detected in breath samples after cannabis inhalation, simultaneous detection of one or more of these cannabinoids might also indicate recent use better than THC alone.

## Data Availability

All data that support the findings of this study are included within the article (and any supplementary files).
